# Effects of Dietary Inositol Supplementation on Growth, Digestive Performance, Antioxidant Capacity, and Body Composition of Golden Pompano (*Trachinotus ovatus*)

**DOI:** 10.3389/fphys.2022.850470

**Published:** 2022-02-11

**Authors:** Xu Chen, Jun Wang, Wei Zhao

**Affiliations:** ^1^Key Laboratory of Aquatic Product Processing, Ministry of Agriculture and Rural Affairs, South China Sea Fisheries Research Institute, Chinese Academy of Fishery Sciences, Guangzhou, China; ^2^Tropical Fisheries Research and Development Center, South China Sea Fisheries Research Institute, Chinese Academy of Fishery Sciences/Sanya Tropical Fisheries Research Institute, Sanya, China; ^3^Southern Marine Science and Engineering Guangdong Laboratory, Zhanjiang, China; ^4^Guangdong Provincial Key Laboratory of Improved Variety Reproduction in Aquatic Economic Animals, School of Life Sciences, Institute of Aquatic Economic Animals, Sun Yat-sen University, Guangzhou, China

**Keywords:** *Trachinotus ovatus*, inositol, growth, digestive performance, antioxidant capacity

## Abstract

A 56-day culture experiment was performed to evaluate effects of inositol supplementation on growth, digestive performance, antioxidant capacity, and body composition of golden pompano (*Trachinotus ovatus*). Five experimental diets (D1, D2, D3, D4, and D5) supplemented with 0, 150, 300, 600, and 1,200 mg kg^−1^ inositol were formulated, respectively. Triplicate groups of 300 fish with an initial weight of (18.78 ± 0.21 g) and 15 seawater cages were used in the feeding experiment. Results indicated that the final body weight (FBW), weight gain rate (WGR), specific growth ratio (SGR), and condition factor (CF) in fish fed with D3–D5 diets were significantly higher than those fed the D1 and D2 diets, and the highest values were detected in D3 diet treatment. The whole-body composition was not significantly affected by different experimental diets. Fish fed with D3-D5 diets showed higher activities of amylase (AMS), lipase (LPS), and superoxide dismutase (SOD), and significantly higher than those fed with D1 and D2 diets. In contrast, fish fed with D3–D5 diets showed lower MDA content and significantly lower than those fed with D1 and D2 diets. The mRNA level of glutathione reductase (GR) in fish fed with D3 and D4 diets was significantly higher than those fed with D1, D2, and D5 diets. Likewise, the mRNA level of catalase (CAT) significantly increased in the dietary inositol groups compared with the D1 group. In conclusion, the supplement of inositol not less than 300 mg kg^−1^ in the diet is indispensable to maintain the rapid growth and promote antioxidative capacity of *T. ovatus*.

## Introduction

Inositol is classified as a B-complex vitamin and is widely distributed in plants and animals in the form of phospholipids as the main structural component of biological membranes (Peres et al., 2004; Cui and Ma, 2020). Biochemical functions of inositol include transmembrane signal transfer, regulation of enzyme activity, mediation of lipid transport and metabolism, and protection of the liver (Wang et al., 2018; Cui and Ma, 2020). The intestinal microbial flora of some species of fish also have a certain ability to synthesize inositol, including Atlantic salmon (*Salmo salar*; Waagbø et al., 1998) and sunshine bass (*Morone chrysops* female × *Morone saxatilis* male; Deng et al., 2002), which do not need dietary inositol supplementation for growth and development. However, for most fish species, their ability to synthesize inositol is not enough to meet the metabolic needs, so dietary inositol supplementation is indispensable for growth and development (Jiang et al., 2010). Moreover, adding enough inositol to the feed is beneficial to the digestion and antioxidant performance of fish (Zhang et al., 2014; Wang et al., 2020). Therefore, inositol is widely supplemented to fish diets as a nutrient. So far, the importance of dietary inositol for maximum growth of fish have been widely reported, such as jian carp (*Cyprinus carpio*; Jiang et al., 2009), gibel carp (*Carassius auratus gibelio*; Gong et al., 2015), parrot fish (*Oplegnathus fasciatus*; Khosravi et al., 2015), Amur sturgeon (*Acipenser schrenckii*; Wang et al., 2018), and taimen (*Hucho taimen* fry; Wang et al., 2020), with the requirement level varying from 100 to 517 mg kg^−1^ diet. In contrast, insufficient supply of dietary inositol can lead to deficiency symptoms, including growth retardation, liver lipid deposition, decreased feed intake, decreased transaminase activity, fin erosion, reduction of skin mucosa and anemia (National Research Council, NRC, 2011).

*Trachinotus ovatus* is widely distributed in southern China, Southeast Asia, Japan, and Australia (Zhao et al., 2021). It has the characteristics of fast growth, high nutritional value, and delicious meat. Due to the increasing market demand, *T. ovatus* has become a very important economically cultured marine fish in the southern coast of China. In order to improve the growth performance and ensure its sustainable supply, it is necessary to optimize feed formula for *T. ovatus*. Up to now, however, no studies have been conducted to investigate the effects of inositol on the growth, digestive performance, antioxidant capacity, and body composition of *T. ovatus*. As the requirement of inositol differs from species to species, the purpose of this study is to determine the requirement for inositol of *T. ovatus*, mainly based on growth, digestive capacity, and antioxidant performance parameters.

## Materials and Methods

### Experimental Diets

Five isonitrogenous and isolipidic diets supplemented with 0 (D1), 150 (D2), 300 (D3), 600 (D4), and 1,200 (D5) mg kg^−1^ inositol (Sigma-Aldrich, Purity ≥ 99%) were prepared for the fish. The formulation and proximate composition analysis of the experimental diets were shown in Table 1. The experimental diets were manufactured following the procedure described by Zhao et al. (2020), and then stored at −20°C until feeding.

**Table 1:** Composition and nutrient levels of the experimental diets.

Items	D1	D2	D3	D4	D5
**Ingredients (g/kg)**					
Fish meal	300.00	300.00	300.00	300.00	300.00
Soybean meal	320.00	320.00	320.00	320.00	320.00
Wheat flour	180.00	179.85	179.70	179.40	178.80
Shrimp head meal	30.00	30.00	30.00	30.00	30.00
Chicken meal	30.00	30.00	30.00	30.00	30.00
Fish oil	65.00	65.00	65.00	65.00	65.00
Soybean lecithin	20.00	20.00	20.00	20.00	20.00
Ca(H_2_PO_4_)_2_	20.00	20.00	20.00	20.00	20.00
Vitamin premix^a^	10.00	10.00	10.00	10.00	10.00
Mineral premix^b^	10.00	10.00	10.00	10.00	10.00
Choline	5.00	5.00	5.00	5.00	5.00
Vc	5.00	5.00	5.00	5.00	5.00
DL-Met	2.50	2.50	2.50	2.50	2.50
Lys-HCL	2.50	2.50	2.50	2.50	2.50
Inositol	0.00	0.15	0.3	0.6	1.2
Total	100.00	100.00	100.00	100.00	100.00
**Nutrient levels^c^ (g/kg)**					
Moisture	101.50	96.10	100.00	94.00	98.60
Crude protein	473.80	472.90	476.50	483.70	480.20
Crude lipid	113.20	115.80	111.70	116.10	114.30
Ash	110.00	112.70	110.00	110.60	114.30
Inositol	0.28	0.40	0.53	0.80	1.39

a Vitamin premix provides the following per kg of diet: vitamin B_1_ 25 mg, vitamin B_2_ 45 mg, vitamin B_6_ 20 mg, vitamin B_12_ 0.1 mg, vitamin K_3_ 10 mg, pantothenic acid 60 mg, niacin 200 mg, folic acid 20 mg, biotin 1.2 mg, retinal acetate 32 mg, vitamin D_3_ 5 mg, vitamin E 120 mg, choline chloride 2.5 g, ethoxyquin 150 mg, and coarse flour 14.012 g.

b Mineral premix provides the following per kg of diet: NaF 2 mg, KI 0.8 mg, CoCl_2_•6H_2_O (1%) 50 mg, CuSO_4_•5H_2_O 10 mg, FeSO_4_•H_2_O 80 mg, ZnSO_4_•H_2_O 50 mg, MnSO_4_•H_2_O 60 mg, MgSO_4_•7H_2_O 1,200 mg, ZnSO_4_•H_2_O 50 mg, Ca(H_2_PO_4_)_2_•H_2_O 3,000 mg, NaCl 100 mg, and zeolite powder 15.447 g.

c Measured values.

### Fish and Feeding Experiment

Juvenile *T. ovatus* were purchased from a commercial farm (Lingshui, Hainan, China). Fifteen seawater cages (1.0 m × 1.0 m × 1.5 m) were placed in the Bay (Lingshui, Hainan, China) and used for feeding experiment. Before the feeding experiment, all fish were acclimatized to the experimental conditions and facilities and fed the experimental control feed for 2 weeks. At the beginning of the feeding experiment, the fish were starved for 24 h, and then healthy fish (average body weight 18.78 ± 0.21 g) were randomly stocked to 15 seawater cages at 20 fish per cage. Each experimental feed was assigned three replicates. The feeding experiment lasted for 56 days, during which fish were slowly hand-fed to apparent satiation twice daily (8:00 and 16:00). At the end of the feeding experiment, all survival fish were starved for 24 h, and then weighed in batches after anesthesia. The final body weight (FBW) and length were gauged to calculate the growth performance.

### Sample Collection

After the feeding experiment, four fish were randomly selected from each cage and frozen in liquid nitrogen, and then stored at −80°C for whole body composition analysis. The liver and mid-gut were stripped from five fish per cage, and frozen in liquid nitrogen and stored at −80°C for enzyme activity analysis. Meantime, the liver was sampled from another three fish per cage, placed in RNAlater™ Stabilization Solution (ThermoFisher Scientific, Shanghai, China) and then stored at −20°C for gene expression analysis.

### Proximate Composition Analysis of the Experimental Diets and Whole Body

The crude lipid, crude protein, moisture, and ash of experimental diets and whole body were determined and analyzed according to the standard procedures of AOAC (1995).

### Enzyme Activity Analysis

Liver and mid-gut samples were homogenized and centrifuged to collect supernatant for enzyme activity analysis using reagent kits (Nanjing Jiancheng Bioengineering Institute, Nanjing, China). Superoxide dismutase (SOD; A001-1) activities and malondialdehyde (MDA; A003-1) contents in liver were determined following the instructions of the kit. Similarly, the activities of amylase (AMS; C016) and lipase (LPS; A054-2) in the mid-gut were measured according to the procedure described by instructions of the kit. The specific operation process is carried out according to the previous description of Zhang et al. (2017).

### Total RNA Extraction and Gene Expression Analysis

Total RNA extraction and real-time quantitative PCR analysis were performed following our previously published methods (Zhao et al., 2020). Briefly, the livers from each cage were pooled for the extraction of total RNA using a Reagent kit (Takara, Dalian, China). Around 10 g/L agarose gel electrophoresis and spectrophotometer (NanoDrop 2000, Thermo Fisher, United States) were used to evaluate the quality and quantity of RNA. Then, cDNA was synthesized using PrimeScript™ RT reagent Kit with gDNA Eraser (Takara, Dalian, China) following the manufacturer’s instructions. Primers for real-time quantitative PCR were presented in Table 2. Real-time quantitative PCR assays were performed on the Light Cycler 480 real-time fluorescence quantitative PCR System (Roche Applied Science, Basel Switzerland). The relative expression levels of target genes were quantified using 2^-ΔΔCt^ method.

**Table 2:** Sequences of primers used for real-time quantitative PCR.

Gene name	Primer sequence (5'-3')	References
*GR*	F-GTGTGTGTGGGCAAGGAGGA R-AGATGAGGTGGGGTGAATGG	Zhao et al., 2020
*CAT*	F-AGTTTTACACCGAGGAGGGC R-TGTGGGTTTGGGGATTGC	Tan et al., 2018
*β-actin*	F-TACGAGCTGCCTGACGGACA R-GGCTGTGATCTCCTTCTGC	Zhao et al., 2020

### Calculations and Statistical Analysis

The weight gain rate (WGR), survival rate (SR), feed conversion ratio (FCR), and specific growth ratio (SGR) were determined according to the equation described by Zhao et al. (2020). Condition factor (*CF*) = 100 × [(body weight, g)/(length, cm)^3^].

All data were presented in the form of means ± SEM. All data were analyzed by one-way ANOVA and Duncan’s multiple range tests in SPSS 19.0 (SPSS, Chicago, IL, United States). A value of *p* < 0.05 was deemed to be statistically significant.

## Results

### Biological Performances

The growth performance (WGR, SGR, and *CF*), SR, and FCR of *T. ovatus* were shown in Table 3. The highest values of FBW, WGR, SGR, and *CF* were detected in fish fed with D3 diet, and significantly higher than those fed the D1 and D2 diets (*p* < 0.05). However, there were no significant difference in the FBW, WGR, SGR, and *CF* among D3–D5 diet treatments (*p* > 0.05). Likewise, SR and FCR were not significantly influenced by dietary treatments (*p* > 0.05).

**Table 3:** Effects of inositol on growth performance of *Trachinotus ovatus*.

Items	IBW/g	FBW/g	WGR/%	SGR/(%/d)	SR/%	FCR	*CF*
D1	18.72 ± 0.30	104.15 ± 7.49^a^	455.32 ± 36.71^a^	2.87 ± 0.19^a^	91.11 ± 7.63	1.04 ± 0.07	3.34 ± 0.24^a^
D2	18.91 ± 0.14	107.95 ± 3.86^a^	470.78 ± 23.05^a^	3.00 ± 0.07^a^	93.33 ± 2.89	1.07 ± 0.10	3.34 ± 0.16^a^
D3	18.68 ± 0.26	123.54 ± 7.26^b^	560.12 ± 37.12^b^	3.23 ± 0.11^b^	90.00 ± 7.32	1.09 ± 0.08	3.66 ± 0.36^b^
D4	18.72 ± 0.27	121.37 ± 7.15^b^	550.02 ± 34.13^b^	3.20 ± 0.16^b^	88.33 ± 5.77	1.05 ± 0.07	3.54 ± 0.30^b^
D5	18.83 ± 0.10	121.02 ± 5.61^b^	550.69 ± 31.19^b^	3.19 ± 0.07^b^	95.00 ± 7.07	1.04 ± 0.08	3.58 ± 0.21^b^

Values are means ± SE of three replicates. The superscript small letters (a,b) in the same column means the significant difference at *p* < 0.05.

### Body Composition

Whole body composition was shown in Table 4. The values of crude protein, crude lipid, moisture, and ash were not significantly affected by different experimental diets (*p* > 0.05).

**Table 4:** Effects of inositol on whole body composition (g/kg dry matter) of *Trachinotus ovatus*.

Items	D1	D2	D3	D4	D5
Moisture	710.4 ± 10.6	713.0 ± 8.9	711.6 ± 14.4	714.3 ± 17.2	703.6 ± 14.3
Crude protein	607.2 ± 16.1	609.5 ± 19.9	623.4 ± 7.3	621.9 ± 16.5	593.0 ± 15.9
Crude lipid	238.4 ± 15.3	246.7 ± 10.7	228.4 ± 14.4	237.3 ± 10.1	251.1 ± 19.2
Ash	156.2 ± 6.1	149.9 ± 4.5	152.3 ± 13.4	155.5 ± 11.4	144.4 ± 12.0

Values are means ± SE of three replicates.

### Antioxidant Capacity

**Figure 1:**
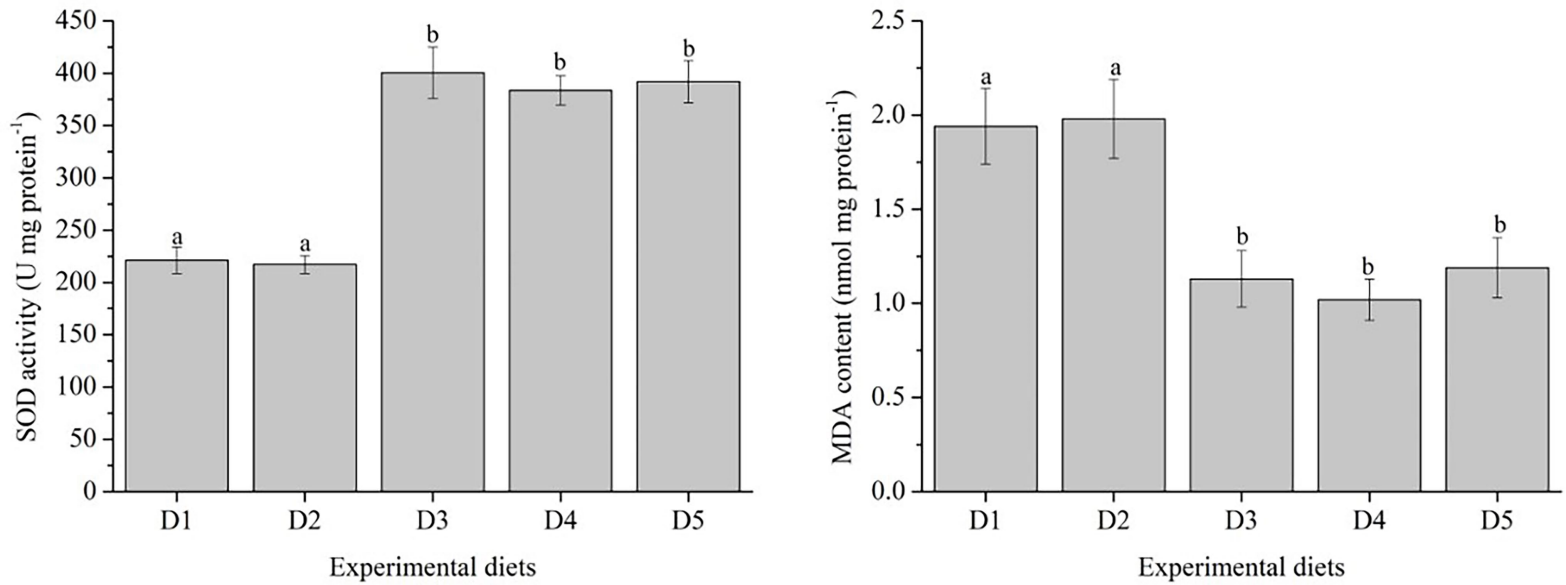
Effects of inositol levels on antioxidant parameters of *Trachinotus ovatus* during the 8 weeks rearing period. *n* = 3. The small letters indicated significant differences at *p*<0.05.

**Figure 2:**
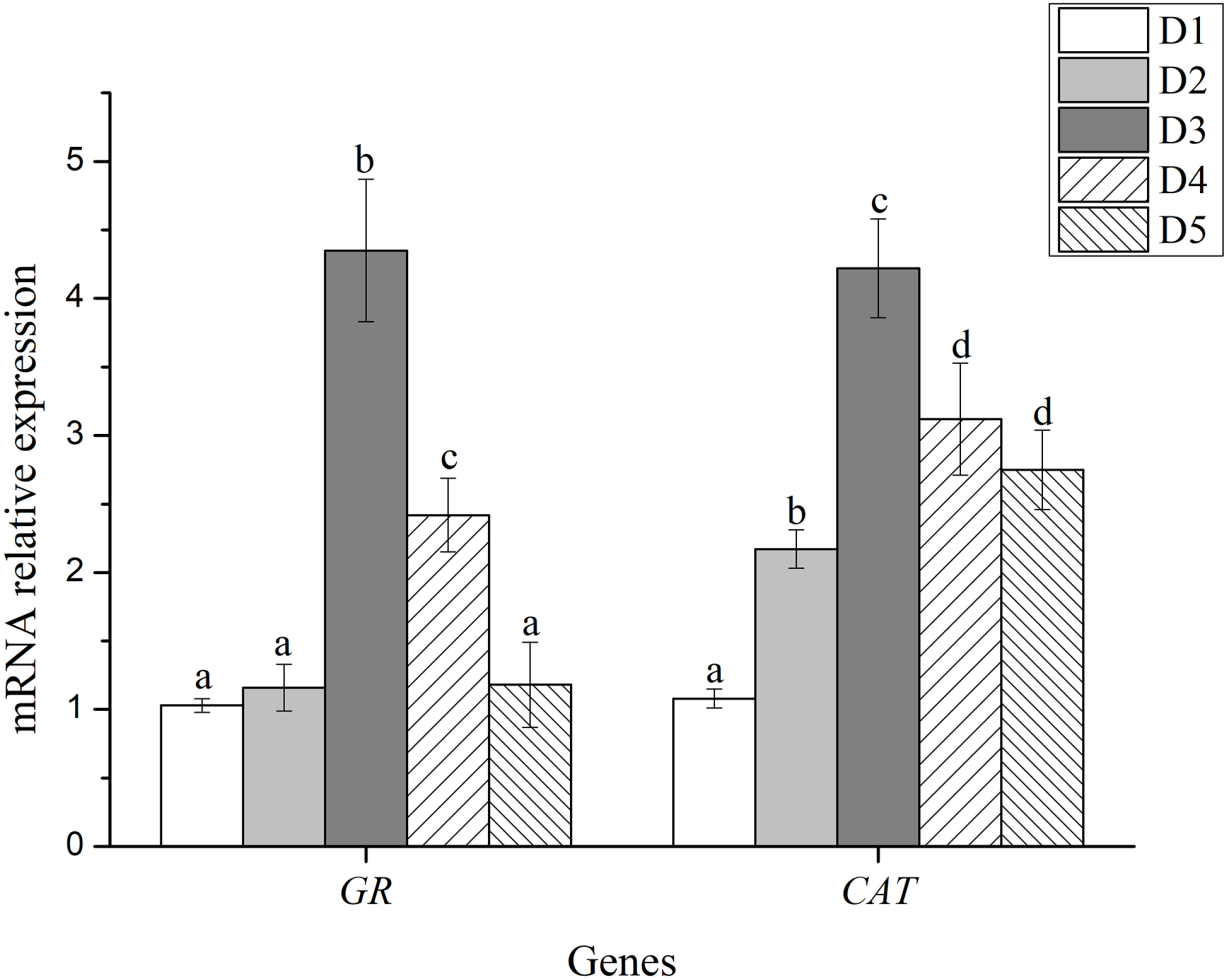
Relative mRNA expression of antioxidant-related genes in liver of fish fed with experimental diets. *n* = 3. The small letters indicated significant differences at *p*<0.05.

Antioxidant enzyme activity and expression levels of antioxidant related-genes were presented in Figures 1, 2, respectively.

The SOD activity in fish fed with D3–D5 diets was significantly higher than those fed with D1 and D2 diets (*p* < 0.05). In contrast, fish fed with D3–D5 diets showed lower MDA content and significantly lower than those fed with D1 and D2 diets (*p* < 0.05).

The mRNA level of glutathione reductase (*GR*) in fish fed with D3 and D4 diets were significantly higher than those fed with D1, D2, and D5 diets (*p* < 0.05), and the highest value was detected in the D3 diet treatment. Likewise, the mRNA level of catalase (*CAT*) significantly increased in the dietary inositol groups compared with the D1 group (*p* < 0.05), and the highest value was detected in the D3 diet treatment (Figure 2).

### Digestive Enzyme Activity

**Figure 3:**
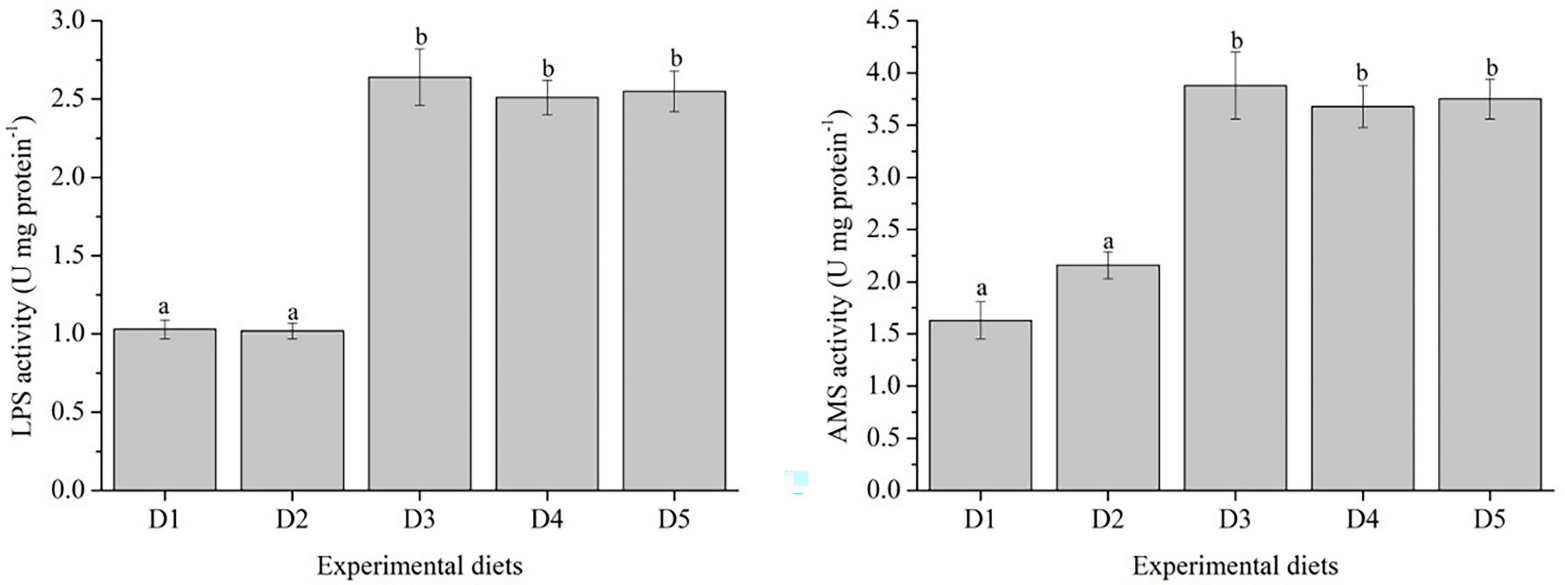
Specific activities of mid-gut digestive enzymes of *Trachinotus ovatus* fed with experimental diets. *n* = 3. The small letters indicated significant differences at *p*<0.05.

The activities of AMS and LPS were shown in Figure 3. Fish fed with D3–D5 diets showed higher activities of AMS and LPS, and significantly higher than those fed with D1 and D2 diets (*p* < 0.05).

## Discussion

A sufficient amount of inositol in the diet to meet the various metabolic needs is extremely important for the fish to obtain the maximum growth. In this study, the growth response of *T. ovatus* to dietary inositol clearly proved the importance of inositol for maximum growth. The growth performance of *T. ovatus* was significantly improved by feeding a diet containing not less than 300 mg kg^−1^ inositol. The similar results were found in studies on *C. carpio* (Jiang et al., 2009), *Lates calcarifer* Bloch (Diao et al., 2010), *C. auratus gibelio* (Gong et al., 2015), *O. fasciatus* (Khosravi et al., 2015), *A. schrenckii* (Wang et al., 2018), and *H. taimen* fry (Wang et al., 2020), which showed that the addition of inositol in the feed could ensure maximum growth and prevent deficiency symptoms. However, Waagbø et al. (1998) and Deng et al. (2002) found that dietary supplementation with inositol did not promote the growth of *S. salar* and sunshine bass (*M. chrysops* × *M. saxatilis*), which indicated that these fish species may be able to synthesize enough inositol for growth without additional supplementation. Current research results demonstrated that the requirement level for inositol in fish diets is related to species differences. In addition, the difference in fish requirement for inositol is also related to growth stage, physiological and nutritional status, source and level of carbohydrate and lipid (Khosravi et al., 2015).

The fluctuation of digestive enzyme activity is a direct response of fish to food source and nutrient concentration (Carneiro et al., 2020). High activity of digestive enzyme helps to improve the digestion and absorption of nutrients, which contributes to the improvement of fish growth performance (Sun et al., 2020; Zhao et al., 2020). Dietary supplementation of inositol improved digestive enzyme activity and promoted growth, which has been reported in studies on *H. taimen* and grass carp (*Ctenopharyngodon idellus*; Zhang et al., 2014; Lin, 2018). Zhang et al. (2014) indicated that the addition of inositol to feed significantly increased the activities of protease and LPS in the intestine of *H. taimen*, which helped to improve the digestion and absorption of feed by fish. Similarly, Lin (2018) also found that inositol supplementation significantly increased the activities of protease, AMS, and LPS in the intestine of *C. idellus*, which was consistent with the results of growth performance. As a structural element of cell membranes, inositol can bind to phospholipids on cell membranes to form inositol phosphate, which helps to maintain the structure and function of the intestine. Therefore, inositol supplementation in feed can promote intestinal development and the secretion of digestive enzymes (Lin, 2018). Similar results were also obtained in this study, the present results indicated that dietary supplementation of inositol not less than 300 mg kg^−1^ significantly increased the activities of AMS and LPS in the intestine of *T. ovatus*. The change trend of growth performance is consistent with that of digestive enzyme activity. Dietary inositol supplementation improved the activity of digestive enzymes to improve the digestion and absorption of nutrients by fish, and finally promote the growth performance of *T. ovatus*.

Regarding body composition, no significant differences were observed in the crude protein, crude lipid, moisture, and ash. The present results indicated that the whole-body composition of *T. ovatus* was not affected by dietary inositol supplementation. The similar results were reported in studies on *S. salar* (Waagbø et al., 1998), *C. idellus* (Wen et al., 2007), and *L. calcarifer* Bloch (Diao et al., 2010). In contrast to our results, Khosravi et al. (2015) found that dietary inositol supplementation significantly reduced the whole-body protein level of *O. fasciatus*. However, dietary inositol supplementation significantly increased the whole-body protein level of *C. auratus gibelio* (Gong et al., 2015). The current results showed that the effect of inositol on whole body composition is species-specific.

Superoxide dismutase, CAT, and GR play important roles in removing oxidative damage and maintaining intracellular homeostasis (Chen et al., 2021). The activation of these antioxidant enzymes is an important protective mechanism for cells to reduce oxidative damage (Zhao et al., 2020). The present results found that dietary supplementation of inositol not less than 300 mg kg^−1^ significantly increased the activity and mRNA level of antioxidant enzyme and decreased MDA content in the liver of *T. ovatus*. Our results demonstrated that dietary inositol supplementation could improve the antioxidant capacity of *T. ovatus* by increasing the activity and mRNA level of antioxidant enzymes. Similarly, Wang et al. (2020) also reported that dietary inositol supplementation significantly increased the SOD activity and decreased MDA content in the skin mucus of *H. taimen*. Dietary supplementation of inositol not less than 232.7 mg kg^−1^ significantly increased the activities of SOD, CAT, and GR in the serum of *C. carpio* (Jiang et al., 2009). Inositol can improve the antioxidant capacity of fish may be related to the structure of polyols.

The polyol structure of inositol can transfer H^+^ to free radicals, scavenge OḤproduced by Feton reaction system, and chelate high-valent chromium compounds, thereby playing an antioxidant effect (Hu et al., 1995; Santoro et al., 2007).

## Conclusion

In conclusion, dietary supplementation of inositol not less than 300 mg kg^−1^ increases the growth performance (WGR, SGR, and *CF*) and digestive enzyme activities (AMS and LPS) of *T. ovatus*, and exerts antioxidant effects by boosting the activities and mRNA levels of antioxidant enzymes. The present results indicated that *T. ovatus* lacks the ability to biosynthesize inositol or the synthesis level of inositol is insufficient to supply the requirements for growth and metabolism. Therefore, the supplement of inositol not less than 300 mg kg^−1^ in the diet is indispensable to maintain the rapid growth and promote antioxidative capacity of *T. ovatus*.

## Data Availability Statement

The raw data supporting the conclusions of this article will be made available by the authors, without undue reservation.

## Ethics Statement

All experimental procedures were conducted in conformity with institutional guidelines for the care and use of laboratory animals in Sun Yat-sen University, Guangzhou, China, and conformed to the National Institutes of Health Guide for Care and Use of Laboratory Animals (Publication No. 85–23, revised 1985).

## Author Contributions

WZ and JW designed the study, analyzed the results, and wrote the paper with contributions from the other authors. XC carried out the rearing work and measured experimental parameters. All authors contributed to the article and approved the submitted version.

## Funding

The study was supported by National Natural Science Foundation of China (32172984); the Southern Marine Science and Engineering Guangdong Laboratory funds (ZJW-2019-06); Central Public-interest Scientific Institution Basal Research Fund, CAFS (2020TD55); and Central Public-interest Scientific Institution Basal Research Fund, South China Sea Fisheries Research Institute, CAFS (2020XK02, 2021SD09).

## Conflict of Interest

The authors declare that the research was conducted in the absence of any commercial or financial relationships that could be construed as a potential conflict of interest.

## Publisher’s Note

All claims expressed in this article are solely those of the authors and do not necessarily represent those of their affiliated organizations, or those of the publisher, the editors and the reviewers. Any product that may be evaluated in this article, or claim that may be made by its manufacturer, is not guaranteed or endorsed by the publisher.
